# Association of microRNAs genes polymorphisms with arthritis: a systematic review and meta-analysis

**DOI:** 10.1042/BSR20190298

**Published:** 2019-07-19

**Authors:** Yingqi Xiao, Hui Liu, Li Chen, Yang Wang, Xiang Yao, Xiaolian Jiang

**Affiliations:** 1 West China School of Nursing/West China Hospital, Sichuan University, Chengdu 610041, Sichuan Province, China; 2 Key Laboratory of Birth Deficits and Related Diseases of Women and Children, West China Second Hospital, Sichuan University, Chengdu 610041, Sichuan Province, China; 3 Department of Orthopedics, West China Hospital, Sichuan University, Chengdu 610041, Sichuan Province, China; 4 Research Department, Childrens Hospital of Chongqing Medical University, Chongqing 401147, China; 5 Department of Radiology, The Xiangan Hospital of Xia Men University, Xiamen 361101, Fujian Province, China

**Keywords:** Arthritis, MicroRNA, polymorphisms

## Abstract

**Objective:** To investigate whether microRNAs genes’ polymorphisms are associated with arthritis. **Methods:** The PubMed, Cochrane Library et al. were systematically searched to identify case–control studies, systematic reviews and meta-analyses. A meta-analysis was performed to calculate odds ratios (ORs), and confidence intervals (CIs) at 95% using fixed-effect model or random-effects model. **Results:** Twenty-two case–control studies involving 10489 participants fulfilled the inclusion criteria. MiR-146a rs2910164 (G/C) was not significantly associated with the risk of rheumatoid arthritis (RA) in any model. Significant associations were found between miR-146a rs2910164 (G/C) and the risk of psoriatic arthritis (PsA) in the heterozygous model and the dominant model. The heterozygous model showed a significant association between the miR-146a rs2910164 (G/C) polymorphism and ankylosing spondylitis (AS). And there was no significant association of miR-146a rs2910164 (G/C) with risk of juvenile rheumatoid arthritis (JRA) at any model. Additionally, there was a significant association of miR-499 rs3746444 (T/C) with risk of RA at two genetic models, and with a moderate heterogeneity. When subgroup analysis by ethnicity, significant associations were almost found between miR-499 rs3746444 (T/C) and the risk of RA in any model in Caucasian populations, and there is no heterogeneity. **Conclusions:** The association of miR-146a rs2910164 (G/C) with RA was not found. And there was a significant association between miR-146a rs2910164(G/C) and PsA or AS. MiR-499 rs3746444 (T/C) was associated with RA in Caucasian populations. These findings did not support the genetic association between miR-146a rs2910164 (G/C) and JRA susceptibility, as well as the association of miR-196a-2 rs11614913 (C/T), miR-146a rs2431697, miR-146a rs57095329, miR-149 rs22928323 with arthritis.

## Introduction

Arthritis is a general term for acute or chronic inflammatory diseases of joint [1]. Common types include diseases such as rheumatoid arthritis (RA), osteoarthritis (OA), psoriatic arthritis (PsA), ankylosing spondylitis (AS), juvenile rheumatoid arthritis (JRA) and other forms of arthritis. Arthritis is characterized by the progression of synovial inflammation leading to joint destruction [2], causing complications such as pain and limited activity, resulting in continuous impairment of physical function and quality of life [3]. Approximately 23% of adults experience arthritis in the United States [4], and arthritis is the most common cause of disability in the past 15 years [5]. In addition, the muscle atrophy of children with JRA progresses rapidly, leading to skeletal developmental disorders, affecting their growth and development [6]. Patients with arthritis may need more social and nursing care. The increased social and economic burdens associated with arthritis worldwide make their targeting treatment a major public health goal.

The etiology of arthritis is unknown, but genetic factors are thought to be important in the pathogenesis and progress [7,8]. MicroRNA (miRNA) is a class of noncoding RNA regulating at least one-third of human protein-encoding genes [9], playing an important role in proliferation, apoptosis, differentiation, immune response and inflammation [10–12]. It was thought that miRNAs fine-tune the immune response and the inflammatory response through the negative feedback loop of the Toll/Interleukin-1 (IL-1) receptor (TIR) signaling downstream molecule [13]. By studying miRNAs among arthritis patients, it was found that aberrant expression of miRNA is associated with arthritis, which is manifested by up-regulation of miRNA concentrations in arthritis patients [14–16]. Single nucleotide polymorphisms (SNPs) presenting in the miRNA gene region may affect the property of miRNA through altering the miRNA expression and/or maturation, leading to aberrant miRNA regulation [17]. Molecular epidemiological studies showed robust evidence that the presence of these genetic polymorphisms would be associated with a variety of diseases, such as inflammatory bowel [18], systemic lupus erythematosus (SLE) [19], some cancers [20] and RA [21,22]. Recently, studies assessing miR-146a rs2910164 or miR-499 rs3746444 polymorphism and risk of RA have been published [23–25], but the results are not always consistent. And other studies add to the evidence base of the associations of other miRNA polymorphism with arthritis risk but are not limited to RA – but not exclusively – the amount and impact of inflammation [24,26]. Therefore, we aimed to perform a systematic review and meta-analysis to systematically evaluate the relationship between miRNA polymorphism and arthritis risk, which can provide a further reference for finding excellent biomarkers and potential therapeutic targets of arthritis.

## Methods

This meta-analysis design was based on the Preferred Reporting Items for Systematic Reviews and Meta-Analyses (PRISMA) prospectively.

### Study selection

A systematic search of the PubMed, Cochrane Library, ISI Web of Science and Embase databases was conducted through May 2018 using the search terms ‘microRNA OR miRNA OR microRNAs’, ‘arthritis’ and ‘polymorphism or variant or mutation or polymorphisms or variants or mutations’, without any languages’ restrictions. The original research of published systematic reviews or meta-analyses were included for further relevant studies. Inclusion criteria of studies for the meta-analyses were published articles: (i) evaluation of the association between microRNAs genes polymorphisms and arthritis; (ii) genotype/allele distributions should be provided for estimating the odds ratios (ORs) and 95% confidence intervals (CIs) and (iii) a case–control design. Exclusion criteria: (i) abstracts, editorial, letters, case reports and other studies which were not focusing on humans; (ii) repeated or overlapping publications; (iii) studies with no detailed genotype distributions or allele data and (iv) family-based studies of pedigrees.

### Data extraction

The data from eligible studies were extracted independently by two researchers based on the inclusion and exclusion criteria. The following information was collected from each study: first author, publication year, country, ethnicity, control source, disease type, genotyping technology, numbers of cases and controls, genotype and allele distributions, Hardy–Weinberg equilibrium (HWE) in control subjects. Disagreements were resolved by consensus or a third researcher.

### Quality score assessment

Study qualities were judged independently by two researchers according to the Newcastle–Ottawa Scale (NOS) (http://www.ohri.ca/programs/clinical_epidemiology/oxford.asp). According to the NOS, study qualities were evaluated based on the following three aspects: selection, comparability, exposure. The NOS uses a star grading system, with the minimum of zero point and the maximum of nine points. The studies graded with >7 stars were considered to be a high-quality study. Disagreements were resolved by consensus or a third researcher.

### Statistical analysis

The associations between microRNAs genes polymorphisms and arthritis was evaluated from the case–control studies using ORs and CIs at 95%. The pooled ORs and CIs were calculated, and their significance was determined by *P*-value to demonstrate the potential relationship with arthritis. When the *P*-value of the Z test was less than 0.05 was considered statistically significant. In our study, five genetic models for each microRNA (allelic models, homozygote model, heterozygous model, recessive model and dominant model) were analyzed. The pooled effect of all subjects based on the disease type for each microRNA were compared. Meta-analysis was performed when more than one study was included in the study.

Heterogeneity among studies was assessed by Chi-squared based Q-test and *I^2^*statistics. If *P* > 0.10 and *I^2^* < 50%, the fixed-effects model (FEM) was applied to estimate pooled OR, else the random-effects model (REM) was used. HWE was assessed for each study by Chi-square test in control groups, and *P*-value <0.05 was considered a significant departure from HWE. In addition, stratified comparisons based on disease were made to further explore the potential heterogeneity according to female, different ethnicity (Asian and Caucasian), control source (hospital-based (HB) and population-based (PB) population), quality score of studies.

To investigate the potential origin of heterogeneity and validate the reliability of this meta-analysis, sensitivity analysis was performed to evaluate the effect of each study on the combined ORs by sequentially excluding individual studies. Potential publication bias was visually assessed by Begg’s funnel plot and statistically determined by Egger linear regression test. Step-down Bonferroni’s test [27] was used for a multiple testing correction for *P*-value estimation and the *P*_corr_-value less than 0.05 was considered statistically significant. All statistical analyses were conducted using Stata version SE 11 (StataCorp LP, College Station, TX).

## Results

### Study inclusion and characteristics

**Figure 1:**
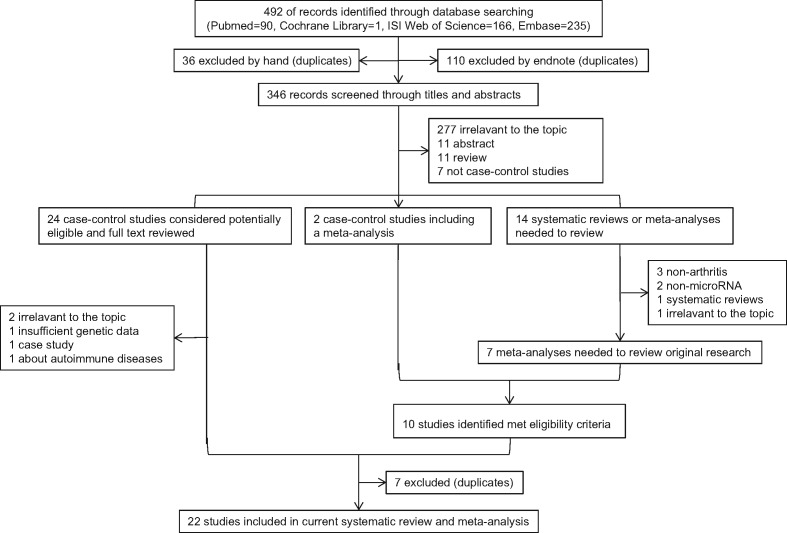
Literature Search and Screening Process

The literature search using databases yielded 492 potentially eligible records, and 40 full texts of these records were reviewed. Of 40 records, 24 records were case–control studies, 14 records were systematic reviews or meta-analyses, and 2 records were case–control studies which included a meta-analysis. After reading the full-text, 21 case–control studies met the inclusion criteria. In addition, one original research from published systematic reviews or meta-analyses was included for our meta-analysis. Supplementary Table S1 summarizes the case–control studies included in the systematic reviews or meta-analyses. The process of study selection was shown in Figure 1. Of the 22 studies that were included in the meta-analyses, there were a total of 10489 participants (4509 cases and 5980 controls) involving six SNPs of microRNAs: miR-146a rs2910164, miR-499 rs3746444, miR-196a-2 rs11614913, miR-146a rs2431697, miR-146a rs57095329, miR-149 rs22928323, and four arthritis: RA, PsA, JRA, AS. Specifically, there were 17 studies on miR-146a rs2910164 (G/C) (including: 11 focusing on RA [23–25,28–35], 2 focusing on PsA [26,36], 2 focusing on JRA [37,38], 2 focusing on AS [8,39]), 10 studies on miR-499 rs3746444 (T/C) (including: 9 focusing on RA [24,25,31,32,34,40–43], 1 focusing on AS [8]), 2 studies on miR-196a-2 rs11614913 (C/T) focusing on RA [24,41], 1 study on miR-146a rs2431697 (T/C) focusing on AS [39], 1 study on miR-146a rs57095329 (A/G) focusing on AS [39], 1 study on miR-149 rs22928323 (T/C) focusing on RA [44]. The characteristics of included studies are shown in Table 1. The distributions of microRNAs genes polymorphisms alleles and genotypes are shown in Table 2.

**Table 1:** Characteristics of 22 included studies in this meta-analysis

Author	Year	Country	Ethnicity	Cases	Control	Control source	Disease	Polymorphisms studied	Quality score
Aleman-Avila	2017	Mexico	Caucasian	412	486	PB	RA	miR-146a rs2910164 (G/C), miR-499 rs3746444(T/C), miR-196a-2 rs11614913 (C/T)	5
Ayeldeen	2018	Egypt	Caucasian	52	56	PB	RA	miR-146a rs2910164 (G/C), miR-499 rs3746444(T/C)	8
Ben Hassine	2017	Tunisia	Caucasian	165	150	PB	RA	miR-146a rs2910164 (G/C)	8
Bogunia-Kubik	2016	Poland	Caucasian	111	130	PB	RA	miR-146a rs2910164 (G/C)	5
Chatzikyriakidou	2010a	Greece	Caucasian	136	147	PB	RA	miR-146a rs2910164 (G/C)	8
Chatzikyriakidou	2010b	Greece	Caucasian	29	66	PB	PsA	miR-146a rs2910164 (G/C)	7
Ciccacci	2016	Italy	Caucasian	192	278	PB	RA	miR-146a rs2910164 (G/C)	8
El-Shal	2013	Egypt	Caucasian	217	245	PB	RA	miR-146a rs2910164 (G/C), miR-499 rs3746444(T/C)	8
Fattah	2018	Egypt	Caucasian	100	100	PB	RA	miR-499 rs3746444(T/C)	8
Hashemi	2013	Iran	Caucasian	104	110	PB	RA	miR-146a rs2910164 (G/C), miR-499 rs3746444(T/C)	7
Jimenez-Morales	2012	Mexico	Caucasian	210	531	PB	JRA	miR-146a rs2910164 (G/C)	7
Maharaj	2018	South Africa	Indian and Caucasian	116	100	PB	PsA	miR-146a rs2910164 (G/C)	8
Niu	2015	China	Asian	611	617	PB	AS	miR-146a rs2910164 (G/C), miR-146a rs2431697 (T/C), miR-146a rs57095329 (A/G)	8
Qian	2012	China	Asian	123	220	HB	RA	miR-146a rs2910164 (G/C)	8
Singh	2014	India	Caucasian	150	216	PB	JRA	miR-146a rs2910164 (G/C)	8
Toraih	2016	Egypt	Caucasian	95	200	PB	RA	miR-499 rs3746444(T/C), miR-196a-2 rs11614913 (C/T)	8
Xiao	2015	China	Asian	186	120	HB	RA	miR-149 rs22928323 (T/C)	7
Xu	2015	China	Asian	102	105	HB	AS	miR-146a rs2910164 (G/C), miR-499 rs3746444(T/C)	7
Yang	2011	China	Asian	208	240	PB	RA	miR-146a rs2910164 (G/C), miR-499 rs3746444(T/C)	8
Yang	2017	China	Asian	386	576	PB	RA	miR-499 rs3746444(T/C)	8
Zhou	2015	China	Asian	598	821	HB	RA	miR-146a rs2910164 (G/C)	8
Zhang	2013	China	Asian	206	466	HB	RA	miR-499 rs3746444(T/C)	8

**Table 2:** Distributions of microRNAs genes polymorphisms alleles and genotypes

Gene Polymorphisms	Author	Year	HWE	Cases GN (%)	Cases CN (%)	Controls GN (%)	Controls CN (%)	Cases GG	Cases GC	Cases CC	Controls GG	Controls GC	Controls CC
miR-146a rs2910164 (G/C)	Aleman-Avila	2017	Yes	532 (64.90)	288 (35.10)	658 (67.70)	314 (32.30)	168	196	46	218	222	46
	Ayeldeen	2018	Yes	50 (48.00)	54 (52.00)	36 (32.10)	76 (67.90)	11	28	13	4	28	24
	Hassine	2017	Yes	251 (76.00)	79 (24.00)	205 (68.00)	95 (32.00)	94	63	8	68	69	13
	Bogunia-Kubik	2016	Yes	176 (79.28)	46 (20.72)	212 (81.54)	48 (18.46)	72	32	7	88	36	6
	Chatzikyriakidou	2010a	Yes	199 (73.16)	73 (26.84)	213 (72.45)	81 (27.55)	73	53	10	80	53	14
	Ciccacci	2016	Yes	287 (74.34)	97 (25.66)	433 (72.65)	163 (27.35)	109	69	14	158	117	23
	El-Shal	2013	No	163 (37.60)	271 (62.40)	149 (30.40)	341 (69.60)	30	103	84	15	119	111
	Hashemi	2013	Yes	153 (73.60)	55 (26.40)	165 (75.00)	55 (25.00)	57	39	8	64	37	9
	Qian	2012	Yes	97 (39.00)	149 (61.00)	179 (41.00)	261 (59.00)	16	65	42	35	109	76
	Yang	2011	Yes	151 (36.30)	265 (63.70)	176 (36.67)	304 (63.33)	28	95	85	30	116	94
	Zhou	2015	Yes	511 (42.73)	685 (57.27)	687 (41.84)	955 (58.16)	114	283	201	151	385	285
	Chatzikyriakidou	2010b	Yes	40 (68.97)	18 (31.03)	96 (72.73)	36 (27.27)	14	12	3	39	18	9
	Maharaj	2018	Yes	149 (64.22)	83 (35.78)	148 (74.00)	52 (26.00)	43	63	10	52	44	4
	Jimenez-Morales	2012	Yes	284 (68.00)	136 (32.00)	701 (66.00)	361 (34.00)	102	80	28	236	229	66
	Singh	2014	Yes	206 (68.66)	94 (31.34)	315 (72.92)	117 (27.08)	75	56	19	112	91	13
	Niu	2015	Yes	496 (41.20)	708 (58.80)	502 (41.10)	718 (58.90)	107	282	213	93	316	201
	Xu	2015	Yes	100 (49.02)	104 (50.98)	73 (34.76)	137 (65.24)	25	50	27	12	49	44

				Cases TN (%)	Cases CN (%)	Controls TN (%)	Controls CN (%)	Cases TT	Cases TC	Cases CC	Controls TT	Controls TC	Controls CC
miR-499 rs3746444 (T/C)	Aleman-Avila	2017	Yes	761 (92.40)	63 (7.60)	910 (93.60)	62 (6.40)	352	57	3	425	60	1
	Toraih	2016	No	115 (60.50)	75 (39.50)	232 (58.00)	168 (42.00)	50	15	30	82	68	50
	Ayeldeen	2018	Yes	49 (47.10)	55 (52.90)	69 (61.60)	43 (38.40)	13	23	16	21	27	8
	El-Shal	2013	Yes	319 (73.50)	115 (26.50)	404 (82.40)	86 (17.60)	113	93	11	167	70	8
	Fattah	2018	Yes	119 (59.50)	81 (40.50)	139 (69.50)	61 (30.50)	33	53	14	49	41	10
	Hashemi	2013	No	124 (59.60)	84 (40.40)	173 (78.60)	47 (21.40)	46	32	26	74	25	11
	Yang	2011	Yes	360 (86.54)	56 (13.46)	417 (86.88)	63 (13.12)	159	42	7	182	53	5
	Yang	2017	Yes	663 (85.90)	109 (14.10)	1011 (87.80)	141 (12.20)	282	99	5	443	125	8
	Zhang	2013	Yes	362 (87.86)	50 (12.14)	802 (86.05)	130 (13.95)	159	44	3	346	110	10
	Xu	2015	Yes	180 (88.24)	24 (11.76)	179 (85.24)	31 (14.76)	79	22	1	74	31	0

				Cases CN (%)	Cases TN (%)	Controls CN (%)	Controls TN (%)	Cases CC	Cases CT	Cases TT	Controls CC	Controls CT	Controls TT
miR-196a-2 rs11614913 (C/T)	Aleman-Avila	2017	Yes	497 (60.30)	327 (39.70)	594 (61.10)	378 (38.90)	142	213	57	182	230	74
	Toraih	2016	No	145 (76.30)	45 (23.70)	290 (72.50)	110 (27.50)	53	39	3	110	70	20
				Cases TN (%)	Cases CN (%)	Controls TN (%)	Controls CN (%)	Cases TT	Cases TC	Cases CC	Controls TT	Controls TC	Controls CC
miR-146a rs2431697 (T/C)	Niu	2015	No	1005 (82.50)	213 (17.50)	1012 (82.10)	220 (17.90)	413	179	17	420	172	24

				Cases AN (%)	Cases GN (%)	Controls AN (%)	Controls GN (%)	Cases AA	Cases AG	Cases GG	Controls AA	Controls AG	Controls GG
miR-146a rs57095329 (A/G)	Niu	2015	Yes	976 (80.30)	240 (19.70)	991 (80.80)	235 (19.20)	391	194	23	404	183	26

				Cases CN (%)	Cases TN (%)	Controls CN (%)	Controls TN (%)	Cases CC	Cases TC	Cases TT	Controls CC	Controls TC	Controls TT
miR-149 rs22928323 (T/C)	Xiao	2015	No	183 (49.20)	189 (50.80)	172 (71.67)	68 (28.33)	44	95	47	74	24	22

### Quantitative data synthesis

We only performed quantitative data synthesis on studies conforming to HWE expectations. Therefore, our study carried out meta-analyses on the inclusion studies of miR-146A rs2910164 (G/C) and miR-499 rs3746444 (T/C), respectively. In addition, we conducted subgroup analyses of the inclusion of miR-146a rs2910164 (G/C), miR-499 rs3746444 (T/C) in accordance with the prior plan. When heterogeneity existed, the REM was applied to calculate the summary ORs in genetic model (Supplementry Table S2). The results of meta-analyses have been summarized in Table 3.

**Table 3:** Summary of overall results and subgroup for the association between the microRNAs genes polymorphisms and arthritis risk

Gene	Groups	Subgroups	Number	Sample size (*n*)		Allelic model			Homozygote model			Heterozygous model			Recessive model			Dominant model		
				Case	Control	OR (95% CI)	POR	POR_corr_	OR (95% CI)	POR	POR_corr_	OR (95% CI)	POR	POR_corr_	OR (95% CI)	POR	POR_corr_	OR (95% CI)	POR	POR_cor_
miR-146a rs2910164 (G/C)	Diseases																			
	RA		10	2101	2638	1.026 (0.941, 1.118)	0.562	0.562	1.056 (0.874, 1.277)	0.573	1.146	1.018 (0.887, 1.168)	0.803	4.015	1.043 (0.900, 1.210)	0.574	1.722	1.026 (0.900, 1.169)	0.702	2.808
		Female	2	644	828	1.050 (0.905, 1.220)	0.518	2.072	1.143 (0.846, 1.544)	0.384	1.152	1.183 (0.887, 1.578)	0.253	0.253	1.006 (0.810, 1.250)	0.955	4.775	1.164 (0.889, 1.525)	0.270	0.540
		***Ethnicity***																		
		Caucasian	7	1172	1357	1.089 (0.898, 1.321)	0.385	0.385	1.094 (0.819, 1.461)	0.543	1.629	1.024 (0.866, 1.211)	0.784	3.920	1.104 (0.842, 1.448)	0.474	0.948	1.069 (0.848, 1.349)	0.572	2.288
		Asian	3	929	1281	1.013 (0.897, 1.144)	0.835	1.670	1.028 (0.800, 1.322)	0.828	0.828	1.005 (0.791, 1.278)	0.965	4.825	1.018 (0.853, 1.216)	0.840	2.520	1.015 (0.809, 1.272)	0.898	3.592
		***Control source***																		
		PB	8	1380	1597	1.066 (0.907, 1.252)	0.438	0.438	1.082 (0.834, 1.403)	0.553	1.106	1.032 (0.878, 1.213)	0.700	2.800	1.043 (0.836, 1.300)	0.711	3.555	1.036 (0.888, 1.207)	0.655	1.965
		HB	2	721	1041	1.020 (0.890, 1.169)	0.772	1.544	1.027 (0.778, 1.356)	0.849	2.547	0.980 (0.753, 1.275)	0.880	3.520	1.044 (0.854, 1.276)	0.673	0.673	1.001 (0.781, 1.282)	0.997	4.985
		***Quality score***																		
		≤7 points	3	627	726	0.886 (0.750, 1.046)	0.154	0.154	0.792 (0.536, 1.170)	0.242	0.726	0.876 (0.697, 1.101)	0.257	1.028	0.845 (0.582, 1.227)	0.375	1.875	0.861 (0.692, 1.071)	0.178	0.356
		>7 points	7	2692	3547	1.083 (0.979, 1.198)	0.123	0.123	1.155 (0.929, 1.435)	0.195	0.585	1.108 (0.933, 1.316)	0.244	0.976	1.086 (0.923, 1.276)	0.320	1.600	1.132 (0.962, 1.332)	0.137	0.274
	PsA	Mixture	2	145	166	0.680 (0.478, 0.967)	0.032	0.096	0.535 (0.220, 1.300)	0.167	0.668	0.567 (0.351, 0.919)	0.021	**0.042**	0.709 (0.302, 1.665)	0.429	2.145	0.570 (0.359, 0.905)	0.017	**0.017**
	JRA	Mixture	2	360	747	0.974 (0.804, 1.181)	0.792	3.960	0.721 (0.332, 1.567)	0.409	1.227	1.179 (0.898, 1.547)	0.236	0.236	0.675 (0.331, 1.379)	0.281	0.562	1.080 (0.838, 1.392)	0.554	2.216
	AS	Asian	2	713	722	1.307 (0.736, 2.319)	0.361	1.444	1.795 (0.592, 5.445)	0.301	0.903	1.378 (1.024, 1.854)	0.034	**0.034**	1.280 (0.586, 2.797)	0.536	2.680	1.081 (0.924, 1.265)	0.192	0.384
miR-499 rs3746444 (T/C)	Diseases																			
	RA		7	1581	2169	0.791 (0.651, 0.962)	0.019	**0.038**	0.585 (0.386, 0.885)	0.011	**0.011**	0.796 (0.626, 1.013)	0.064	0.320	0.662 (0.444, 0.987)	0.043	0.172	0.771 (0.603, 0.987)	0.039	0.117
		Female	2	425	485	0.747 (0.459, 1.216)	0.241	0.723	0.541 (0.260, 1.126)	0.100	0.100	0.742 (0.348, 1.582)	0.440	2.200	0.624 (0.302, 1.289)	0.203	0.406	0.718 (0.358, 1.443)	0.352	1.408
		***Ethnicity***																		
		Caucasian	4	781	887	0.656 (0.541, 0.795)	0.000	**<0.01**	0.418 (0.242, 0.722)	0.002	**0.008**	0.645 (0.508, 0.820)	0.000	**<0.01**	0.532 (0.319, 0.885)	0.015	0.075	0.618 (0.490, 0.780)	0.000	**<0.01**
		Asian	3	800	1282	0.962 (0.799, 1.159)	0.686	2.058	0.973 (0.497, 1.904)	0.935	3.740	0.956 (0.773, 1.182)	0.677	1.354	0.974 (0.498, 1.902)	0.938	4.690	0.956 (0.778, 1.175)	0.670	0.670
		***Control source***																		
		PB	6	1375	1703	0.747 (0.646, 0.863)	0.000	**<0.01**	0.514 (0.329, 0.803)	0.003	**0.012**	0.750 (0.631, 0.892)	0.001	**0.003**	0.601 (0.329, 0.921)	0.019	0.095	0.730 (0.617, 0.864)	0.000	**<0.01**
		HB	1	206	466	1.174 (0.828, 1.664)	0.369	0.369	1.532 (0.416, 5.642)	0.521	2.084	1.149 (0.773, 1.708)	0.493	1.479	1.484 (0.404, 5.449)	0.552	2.760	1.173 (0.798, 1.726)	0.417	0.834
		***Quality score***																		
		≤7 points	1	412	486	0.823 (0.572, 1.184)	0.294	0.882	0.276 (0.029, 2.666)	0.266	0.266	0.872 (0.591, 1.286)	0.490	2.450	0.281 (0.029, 2.713)	0.273	0.546	0.842 (0.574, 1.235)	0.379	1.516
		>7 points	6	1169	1683	0.784 (0.622, 0.988)	0.039	0.078	0.603 (0.395, 0.921)	0.019	**0.019**	0.779 (0.582, 1.043)	0.094	0.470	0.684 (0.455, 1.029)	0.068	0.272	0.754 (0.560, 1.015)	0.063	0.189
	AS	Asian	1	102	105	1.299 (0.733, 2.301)	0.370	1.110	0.356 (0.014, 8.869)	0.529	2.645	1.504 (0.800, 2.829)	0.205	0.205	0.321 (0.013, 7.964)	0.488	1.952	1.439 (0.770, 2.690)	0.254	0.508

As the ‘Statistical analysis’ section described, *P*_corr_ value less than 0.05 was considered statistically significant. Abbreviations: miR-146a rs2910164 (G/C), Allelic model (G vs. C); Homozygote model, (GG vs. CC); Heterozygous model, (GG vs. GC); Recessive model, (GC+GG vs. CC); Dominant model, (GG vs. CC+GC); miR-499 rs3746444 (T/C), Allelic model (T vs. C); Homozygote model: (TT vs. CC); Heterozygous model, (TT vs. TC); Recessive model, (TT+TC vs. CC); Dominant model, (TT vs. TC+CC).

### Genetic association for miR-146a rs2910164 (G/C)

**Figure 2:**
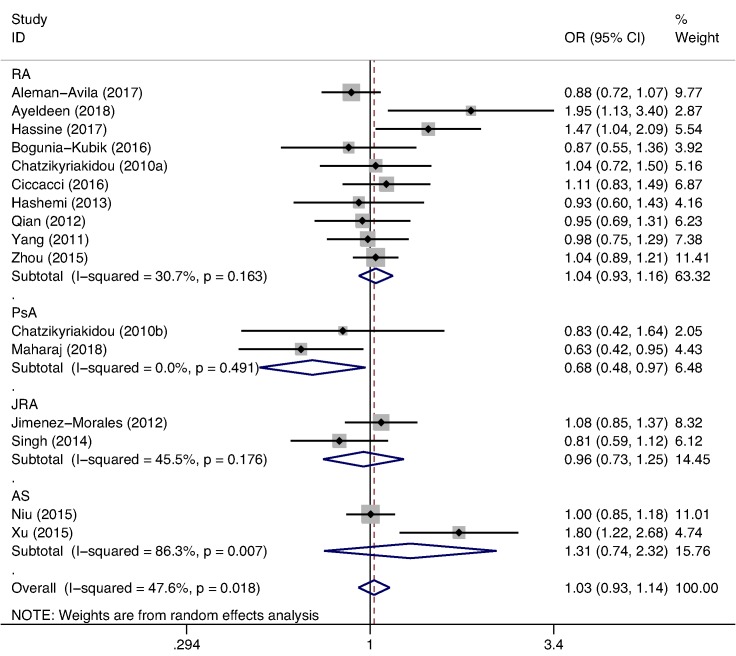
Forest plot of arthritis risk associated with miR-146a rs2910164(G/C)polymorphism in allelic model (G versus C) for the analysis by diseases

After excluding a study [31] that did not conform to HWE expectations, we analyzed the association between miR-146a rs2910164 (G/C) polymorphism and the susceptibility to arthritis in 16 studies with a combined number of 3319 cases and 4273 controls. Based on prior planning, our disease-based analyses showed that association of miR-146a rs2910164 (G/C) polymorphism with PSA or AS susceptibility was identified. Specifically, significant associations between miR-146a rs2910164 (G/C) polymorphism and PsA were found in the allelic model (OR = 0.680, 95% CI = 0.478–0.967, *P*=0.032) (Figure 2), the heterozygous model (OR = 0.567, 95% CI = 0.351–0.919, *P*=0.021), and the dominant model (OR = 0.570, 95% CI = 0.359–0.905, *P*=0.017). Corrected *P*-values for multiple testing remained significant in the heterozygous model and the dominant model. Although there was no statistical association between miR-146a rs2910164 (G/C) polymorphism and PsA in the homozygote model (OR = 0.535, 95% CI = 0.220–1.300, *P*=0.167) and recessive model (OR = 0.709, 95% CI = 0.302–1.665, *P*=0.429), a trend of decreased risk could be seen. Only the heterozygous model showed a significant association between the miR-146a rs2910164 (G/C) polymorphism and AS (OR = 1.378, 95% CI = 1.024–1.854, *P*=0.034) and corrected *P*-values for multiple testing remained significant. However, there was no significant association between miR-146a rs2910164 (G/C) polymorphism and RA or JRA at any genetic model (Table 3). Additionally, to estimate the heterogeneity in the outcomes and the inference of the studies, we pooled the ORs and 95% CI of RA from further subgroup analyses of female, ethnicity, control source and quality score. Statistical significance was not found in any groups in any model. This further strengthened the result that there was no association between miR-146a rs2910164 (G/C) polymorphism and RA.

### Genetic association for miR-499 rs3746444 (T/C)

**Figure 3:**
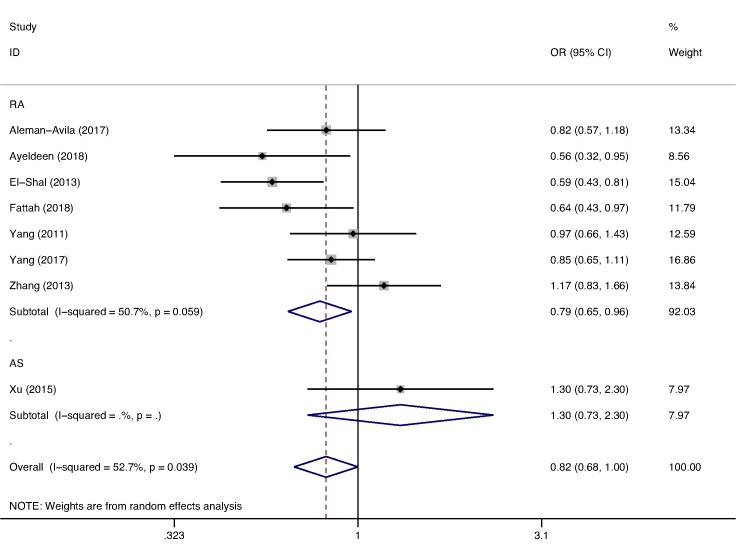
Forest plot of arthritis risk associated with miR-499 rs3746444(T/C)polymorphism in allelic model (T versus C) for the analysis by diseases

After excluding two studies [32,41] that did not conform to HWE expectations, the miR-499 rs3746444 (T/C) polymorphism involved eight studies with a combined number of 1683 cases and 2274 controls. Seven studies with a combined number of 1581 cases and 2169 controls evaluated the miR-499 rs3746444 (T/C) polymorphism and its association with RA, while only one study reported the miR-499 rs3746444 (T/C) polymorphism and its association with AS. The results after a multiple testing correction showed that a significant association between miR-499 rs3746444 (T/C) polymorphism and decreased RA risk was seen in two genetic models (allelic model: OR  =  0.791, 95% CI  =  0.651–0.962, *P*=0.019, *P*_corr_=0.038 (Figure 3); homozygote model: OR  =  0.585, 95% CI  =  0.386–0.885, *P*=0.011, *P*_corr_ =0.011). Based on the female, a significant association between miR-499 rs3746444 (T/C) polymorphism and RA was not found under all the genetic models (Table 3). When subgroup was defined by ethnicity, we were surprised to find that, regardless of the models, miR-499 rs3746444 (T/C) polymorphism was significantly associated with RA in Caucasian population, and corrected *P*-values for multiple testing remained significant in four models (allelic model: OR  =  0.656, 95% CI  =  0.541–0.795, *P*<0.001, *P*_corr_<0.01; homozygote model: OR  =  0.418, 95% CI  =  0.242–0.722, *P*=0.002, *P*_corr_=0.008; heterozygous model: OR  =  0.645, 95% CI  =  0.508–0.820, *P*<0.001, *P*_corr_<0.01; dominant model: OR  =  0.618, 95% CI  =  0.490–0.780, *P*<0.001, *P*_corr_<0.01), but not statistically associated with RA in the Asian population in any of the models. When stratified by control source or quality score, six studies reported the miR-499 rs3746444 (T/C) polymorphism and its association with RA in PB group or quality score >7 points group (Table 3). There was a distinctly decreased overall risk of RA under the allelic and genotypic models in PB group, and corrected *P*-values for multiple testing remained significant in four models (allelic model: OR  =  0.747, 95% CI  =  0.646–0.863, *P*<0.001, *P*_corr_<0.01; homozygote model: OR  =  0.514, 95% CI  =  0.329–0.803, *P*=0.003, *P*_corr_=0.012; heterozygous model: OR  =  0.750, 95% CI  =  0.631–0.892, *P*= 0.001, *P*_corr_=0.003; dominant model: OR  =  0.730, 95% CI  =  0.617–0.864, *P*<0.001, *P*_corr_<0.01). Additionally, after a multiple testing miR-499 rs3746444 (T/C) polymorphism was significantly associated with RA in quality score >7 points group at homozygote model (OR  =  0.603, 95% CI  =  0.395–0.921, *P*=0.019, *P*_corr_=0.019).

### Publication bias

Publication bias was assessed by performing Begg’s funnel plot and Egger’s regression intercept test under all genetic models. Because there are few inclusion studies for other diseases, we actually only conducted publication bias tests on the included studies of the studies of RA. For miR-146a rs2910164 (G/C) and miR-499 rs3746444 (T/C), symmetrical funnel plots were obtained in all the genetic models (Supplementary Figures S1 and S2 showed the result of allelic model). Moreover, the results of Egger’s test (miR-146a rs2910164 (G/C): allelic model: *P*=0.416, homozygote model: *P*= 0.387, heterozygous model: *P*= 0.418, recessive model: *P*=0.558, dominant model: *P*= 0.348; miR-499 rs3746444 (T/C): allelic model: *P*=0.881, homozygote model: *P*=0.532, heterozygous model: *P*=0.898, recessive model: *P*=0.624, dominant model: *P*=0.853) further provided no evidence of significant publication bias.

### Sensitivity analysis

In order to determine the stability of our results, the sensitivity analyses were performed on miR-146a rs2910164 (G/C) and miR-499 rs3746444 (T/C) (Supplementary Figures S3 and S4 showed the result of allelic model). First, the REM was compared with the FEM, and the statistically similar results were acquired under all genetic models. Second, we applied sensitivity analyses by sequentially excluding individual studies to evaluate the influence of individual study on the obtained conclusions. The analyses showed that the conclusions remained unchanged after exclusion of each study, which suggested that all the results were statistically robust.

## Discussion

In this systematic review and meta-analysis, we reviewed the available case–control literature on genetic studies of miRNA SNPs in arthritis and conducted independent meta-analyses for the eligible research. The present study included 22 studies involving six SNPs of miRNA: miR-146a rs2910164 (G/C), miR-499 rs3746444 (T/C), miR-196a-2 rs11614913 (C/T), miR-146a rs2431697 (T/C), miR-146a rs57095329 (A/G), miR-149 rs22928323 (T/C), and four arthritis: RA, PsA, JRA, AS. We carried out a meta-analysis on the inclusion studies of miR-146a rs2910164 (G/C), miR-499 rs3746444 (T/C), respectively.

Because there were many types of arthritis and the pathogenesis may not be the same, analyses based on disease were performed to investigate the relationships between genetic polymorphisms and different arthritis. Our results showed that miR-146a rs2910164 (G/C) was not significantly associated with susceptibility to RA, which was consistent with previous meta-analyses [35,45–50]. However, the result was not consistent with the result of functional studies. Previous studies have found that the levels of miR-146a in RA patients are strongly up-regulated in a variety of tissue structures when compared with non-RA populations [15,51–53]. Up-regulation of miR-146a expression may result in prolonged tumor necrosis factor (TNF)-α (one of inflammatory cytokines) production [15]. It is well known that persistent inflammation is one of the characteristics of the pathogenesis of RA [54]. Therefore, the inconsistency between genetic studies and functional studies makes it difficult to judge the relationship between miR-146a and RA. One of the possible reasons for such result is that the pathogenesis of RA is complex and is the result of the interaction of genes, environment and other parties [55]. It is difficult to explain this association in a single genetic study. When our study population focused on females, the results also showed that no significant association was found between miR-146a rs2910164 (G/C) and RA in all genetic models. However, Zhou et al. [35] reported that miR-146a rs2910164 (G/C) polymorphism was associated with RA in female population, with the heterozygote CT having a more severe and more active form of disease compared with other genotypes. In Zhou et al.’s study [35], they found that genotype GG was significantly associated with RA in the female population and negatively correlated with RA in the male population. The reason for this inconsistency was that a study [31] that did not comply with HWN expectations was included in Zhou et al.’s study [35], which was excluded from our meta-analysis. We conducted an additional analysis to incorporate the present study into meta-analysis, and such association was indeed discovered, but there was a large heterogeneity in three genetic models. However, the heterogeneity in our study was small and was an acceptable range. Additionally, miRNA (miR-146a) is encoded on a non-X chromosome [56]. Khalifa et al. [56] reported that no differences were observed in the expression levels of miR-146a between women and men in RA and healthy subjects. This report strengthened the persuasiveness of our results. Therefore, based on the meta-analyses of available evidence, we still believe that miR-146a rs2910164 (G/C) polymorphism and RA have no significant association. In addition, this is the first meta-analysis reporting that association between miR-146a rs2910164 (G/C) and PsA or AS or JRA. Specifically, a significant decreased risk of PsA was observed in heterozygous, dominant comparison. Association between miR-146a rs2910164 (G/C) and AS was statistically different in comparison of heterozygous model. In addition, we found no significant association in all genetic models between miR-146a rs2910164 (G/C) and JRA. However, the studies included in these comparisons were limited and more research are required to increase statistical power and further confirm whether such differences exist. It is worth mentioning that in addition to considering the changes in the results of statistical power, we should also understand that although RA, PsA, AS or JRA are inflammatory arthritis, their pathogenesis is not completely consistent, and after mutation, the process of regulation in the progression of the disease that miRNA participates may be different, which may therefore produce different results.

Previous studies have shown that miR-499 can regulate the production of anti-cyclic citrullinated peptide antibodies by regulating the expression of its target gene (*PADI4* gene) [57], thereby affecting the production of C-reactive protein and inflammation in RA [40]. In the meta-analyses reported here, we found that in the overall populations there was association between miR-499 rs3746444 (T/C) and RA susceptibility in allelic model and homozygote model, respectively, which can reflect the results of functional studies [57]. And the results are similar to early meta-analyses [46,48,49,58], but is not consistant with Yang et al. [42]. The reason for the inconsistency may be that only one study was statistically significant in the Yang et al.’s inclusion studies and the sample size was small, which have increased the probability of larger *P*-values and wider CIs. Further subgroup analyses showed there was significant association detected in four genetic models (allelic model, homozygote model, heterozygous model, dominant model) among Caucasians, while no significant associations were found in all genetic models among Asians, which was similar to previous meta-analyses [42,46,48,58], suggesting miR-499 rs3746444 (T/C) was associated with RA in Caucasians, but not associated in Asians. In addition, statistical significance was detected in the same four models comparisons in PB group, which makes our results consistent and robust. In >7 points group, miR-499 rs3746444 (T/C) was associated with RA susceptibility in homozygote model, and a trend of decreased susceptibility of RA still existed in other the genetic models. Therefore, the present study suggested that miR-499 rs3746444 (T/C) is one of various microRNAs gene polymorphism, which is an independent factor associated with RA. Thus, small variation in miR-499 rs3746444 may have a meaningful effect on the progression of RA’s autoimmune inflammation and lead to functional consequences.

The present study has several limitations. First, the studies included in the present study were not always high quality, but with subgroup analysis based on quality scores, the results of high quality were almost always consistent with the results of ungrouped studies. Second, the present study divided the race into Caucasian and Asian populations, which may lead to selection bias. Third, we did not analyze factors such as environmental variables, gene×gene and gene×environment interactions due to lack of data. Finally, including studies of sample size in the meta-analysis were relatively small and, as such, our analysis may have been underpowered. However, compared with the previously published systematic reviews, we searched the databases more comprehensively and evaluated the quality of the literature. The diseases included were not limited to single disease such as RA. And to show the robustness of the conclusions, we performed multiple testing corrections technique for *P*-value estimation. In addition, this article not only updates the literature on the relationship between miR 146a rs2910164 (G/C) or miR 499 rs3746444 (T/C) polymorphisms and RA. In addition, this is the first meta-analysis to report the association between miR-146a rs2910164 (G/C) and PsA or AS or JRA. Therefore, this article can further enhance our understanding of the relationship between microRNAs gene polymorphism and arthritis.

## Conclusions

In this meta-analysis of case–control studies, the association of miR-146a rs2910164 (G/C) with RA was not found. And there was a significant association between miR-146a rs2910164(G/C) and PsA or AS. We also found that miR-499 rs3746444 (T/C) was associated with RA, especially in Caucasian populations. Additionally, our results did not support the genetic association between miR-146a rs2910164 (G/C) and JRA susceptibility.

## Supporting information

**Supplementary Figure S1:** Begg’s funnel plot of publication bias test for the association between miR-146a rs2910164 (G/C) polymorphism and RA risk under allelic model (G versus C).

**Supplementary Figure S2:** Begg’s funnel plot of publication bias test for the association between miR-499 rs3746444 (T/C) polymorphism and RA risk under allelic model (T versus C).

**Supplementary Figure S3:** Sensitivity analysis of association of miR-146a rs2910164 (G/C) polymorphism and RA risk under allelic model (G versus C).

**Supplementary Figure S4:** Sensitivity analysis of association of miR-499 rs3746444 (T/C) polymorphism and RA risk under allelic model (T versus C).

**Supplementary Table S1:** Case-control Studies Included In Systematic Reviews and Meta-Analyses. Evaluating association of microRNAs genes polymorphisms with arthritis.

**Supplementary Table S2:** Summary of the P value for heterogeneity test in this meta-analysis.

## Abbreviations

AS: ankylosing spondylitis
CI: confidence interval
FEM: fixed-effects model
HWE: Hardy–Weinberg equilibrium
JRA: juvenile rheumatoid arthritis
miRNA: microRNA
NOS: Newcastle–Ottawa Scale
OR: odds ratio
PB: population-based
PsA: psoriatic arthritis
RA: rheumatoid arthritis
REM: random-effects model
SNP: single nucleotide polymorphism
