# Supplementary material for: Association of microRNAs genes polymorphisms with arthritis: a systematic review and meta-analysis
Source: Biosci Rep. 2019 Jul 19;39(7):BSR20190298. doi: 10.1042/BSR20190298 (PMC6639462; doi:10.1042/BSR20190298)
Supplement: Supplementary file 1 [file bsr20190298_Supp1.pdf]

Supplementary Content

eTable 1. Case-control Studies Included In Systematic Reviews and Meta-Analyses. Evaluating association of microRNAs genes polymorphisms with arthritis.

eTable 2. Summary of the P value for heterogeneity test in this meta-analysis.

eFigure 1. Begg's funnel plot of publication bias test for the association between miR-146a rs2910164 (G/C) polymorphism and RA risk under allelic model (G versus C).

eFigure 2. Begg's funnel plot of publication bias test for the association between miR-499 rs3746444 (T/C) polymorphism and RA risk under allelic model (T versus C).

eFigure 3. Sensitivity analysis of association of miR-146a rs2910164 (G/C) polymorphism and RA risk under allelic model (G versus C).

eFigure 4. Sensitivity analysis of association of miR-499 rs3746444 (T/C) polymorphism and RA risk under allelic model (T versus C).

eTable 1 Case-control Studies Included In Systematic Reviews and Meta-Analyses. Evaluating association of microRNAs genes polymorphisms with arthritis.

Gene Polymorphisms	Disease	Studies	Systematic reviews and meta-analyses									
			Chen 2013	Li 2014	Lu 2014	Yang 2014	Lee 2015	Li 2015	Song 2015	Zhou 2015	Fu 2016	Yang 2017
miR 146a rs2910164	RA	Chatzikyriakidou 2010a[1]	Y	Y		Y	Y		Y	Y	Y	
		Yang 2011[2]	Y	Y		Y	Y		Y	Y	Y	
		Jimenez-Morales 2012[3]	Y	Y		Y				Y	Y	
		Hashemi 2012[5]		Y		Y			Y	Y	Y	
		Qian 2012[4]	Y	Y		Y			Y	Y	Y	
		El-Shal 2013[6]		Y					Y	Y	Y	
		Zhou 2015[7]								Y		
miR 499 rs3746444	RA	Chatzikyriakidou 2010a										
		Yang 2011		Y	Y	Y			Y		Y	Y
		Jimenez-Morales 2012										
		Hashemi 2012		Y	Y	Y			Y		Y	
		Qian 2012										
		El-Shal 2013		Y	Y				Y		Y	Y
		Zhang 2013			Y	Y						Y
miR 146a	PsA	Chatzikyriakidou	Y						Y			

rs2910164		2010b		
miR 146a rs2910164	JRA	Jimenez-Morales 2012	Y	
miR 146a rs2910164	JIA	Singh 2014		Y
		Jimenez-Morales 2012		Y

Abbreviation: Y, yes (Each “Y” indicates that this study was included in the systematic reviews and meta-analyses of corresponding column).

References

eTable 2. Summary of the P value for heterogeneity test in this meta-analysis.

Gene	Groups	Subgroups	No.	Sample size (N)		Allelic model		Homozygote model		Heterozygous model		Recessive model		Dominant model	
				case	control	I2(%)	PHet	I2(%)	PHet	I2(%)	PHet	I2(%)	PHet	I2(%)	PHet
miR-146a rs2910164（G/C）	Diseases														
	RA		10	2101	2638	30.70%	0.163	17.60%	0.281	0.00%	0.489	0.00%	0.624	21.00%	0.249
		Female	2	644	828	0.00%	0.942	0.00%	0.844	0.00%	0.598	0.00%	0.744	0.00%	0.686
		Ethnicity													
		Caucasian	7	1172	1357	52.40%	0.050	42.50%	0.108	21.20%	0.268	9.50%	0.356	43.90%	0.098
		Asian	3	929	1281	0.00%	0.863	0.00%	0.803	0.00%	0.659	0.00%	0.867	0.00%	0.705
		Control source													
		PB	8	1380	1597	45.00%	0.079	33.00%	0.165	9.50%	0.356	1.60%	0.417	34.80%	0.151
		HB	2	721	1041	0.00%	0.623	0.00%	0.508	0.00%	0.430	0.00%	0.905	0.00%	0.429
		Quality score													
		≤7 points	3	627	726	0.00%	0.973	0.00%	0.877	0.00%	0.978	0.00%	0.858	0.00%	0.993
		>7 points	7	2692	3547	32.40%	0.181	25.20%	0.236	0.00%	0.438	0.00%	0.496	21.00%	0.269
	PsA		2	145	166	0.00%	0.491	33.00%	0.222	0.00%	0.901	32.00%	0.225	0.00%	0.743
	JRA		2	360	747	45.50%	0.176	66.10%	0.086	0.00%	0.654	63.10%	0.100	0.00%	0.371
	AS		2	713	722	86.30%	0.007	83.60%	0.013	9.80%	0.292	83.80%	0.013	68.70%	0.074
miR-499 rs3746444	Diseases														

（T/C）															
	RA					50.7%%	0.059	0.00%	0.537	51.50%	0.054	0.00%	0.658	56.50%	0.032
		Female	2	425	485	73.80%	0.051	0.00%	0.756	84.20%	0.012	0.00%	0.964	82.90%	0.016
		Ethnicity													
		Caucasian	4	781	887	0.00%	0.514	0.00%	0.891	29.5%%	0.235	0.00%	0.735	25.30%	0.260
		Asian	3	800	1282	4.40%	0.351	0.00%	0.598	18.50%	0.293	0.00%	0.592	14.20%	0.312
		Control source													
		PB	6	1375	1703	25.1%%	0.246	0.00%	0.752	42.40%	0.123	0.00%	0.778	44.00%	0.112
		HB	1	206	466	/	/	/	/	/	/	/	/	/	/
		Quality score													
		≤7 points	1	412	486	/	/	/	/	/	/	/	/	/	/
		>7 points	6	1169	1683	58.8%%	0.033	0.00%	0.462	59.00%	0.032	0.00%	0.613	63.40%	0.018
	AS		1	102	105	/	/	/	/	/	/	/	/	/	/

Abbreviation: PHet: the P value for heterogeneity test
